# Synergistic Effects of Additive Engineering in Enhancing the Performance of Sn–Pb Perovskite Thin‐Film Transistors and Derived Logic Circuits

**DOI:** 10.1002/advs.202520241

**Published:** 2026-02-04

**Authors:** Zeeshan Alam Ansari, Abhishek Kumar, Soumallya Banerjee, Chintam Hanmandlu, Anjali Thakran, Yu‐Te Chen, Po‐Yu Yang, Shenghan Li, Chun‐Wei Pao, Yun‐Chorng Chang, Chu‐Chen Chueh, Chih‐Wei Chu

**Affiliations:** ^1^ Department of Physics National Taiwan University Taipei Taiwan ROC; ^2^ Research Center for Applied Sciences Academia Sinica Taipei Taiwan ROC; ^3^ Nano‐Science and Technology Program Taiwan International Graduate Program Academia Sinica Taipei Taiwan ROC; ^4^ Department of Chemical Engineering National Taiwan University Taipei Taiwan ROC; ^5^ Department of Photonics National Yang Ming Chiao Tung University Hsinchu City Taiwan ROC

**Keywords:** additive engineering, complementary logic circuits, defect passivation, mixed Sn‐Pb perovskites, thin‐film transistor

## Abstract

Solution‐processed metal halide perovskite transistors possess intrinsic characteristics that hold promise for integration with n‐type semiconductors such as fullerene (C_60_) in CMOS‐like circuits. Yet, their performance and stability remain inferior to n‐type counterparts due to inefficient in‐plane charge transport and defect‐induced instabilities. This study proposes a rational additive engineering strategy using 4,8‐dihydrobenzo[1,2‐b:4,5‐b′]dithiophen‐4,8‐dione (BDTD) to regulate nucleation and crystallization of MA_0.4_FA_0.6_Sn_0.5_Pb_0.5_I_3_ films. BDTD alleviates microstrain, suppresses Sn^4+^‐related defects, and passivates undercoordinated Sn and Pb ions, forming smoother films with enlarged grains. Compared to control devices, the optimized transistor achieves an increase by an order of magnitude in hole mobility (4.1 vs. 0.38 cm^2^ V^−1^ s^−1^) and a substantially improved on/off ratio (1.8 × 10^5^ vs. 3.1 × 10^4^). Moreover, the BDTD‐treated transistors exhibit excellent reproducibility and operational stability under inert conditions without encapsulation. Furthermore, surface passivation using tetrabutylammonium hexafluorophosphate (TBAPF6) reduces interfacial traps, improving reliability and lowering the threshold voltage from 9.89 to 3.6 V. Finally, integration with an n‐type C60 transistor yields a functional perovskite–C60 inverter, demonstrating strong potential for complementary logic applications. This work highlights the synergistic role of additive and interfacial engineering in overcoming intrinsic limitations of Sn‐Pb perovskites, offering a viable pathway toward practical perovskite‐based complementary electronics.

## Graphical abstract

An additive engineering strategy employing 4,8‐dihydrobenzo[1,2‐b:4,5‐b′]dithiophen‐4,8‐dione (BDTD) is utilized to passivate bulk defects, regulate crystallization dynamics, and suppress Sn^4+^ formation in Sn–Pb perovskite thin‐film transistors. The incorporation of BDTD yields a substantial enhancement in electrical performance, increasing the hole mobility from 0.38 cm^2^ V^−1^ s^−1^ (control) to 4.1 cm^2^ V^−1^ s^−1^ (BDTD‐treated) and improving the on/off ratio from 3.1 × 10^4^ to 1.8 × 10^5^.

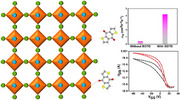

## 1 Introduction

Organic‐inorganic metal halide perovskites (OMHPs) have rapidly emerged as a versatile class of semiconductors, delivering remarkable advances across optoelectronic applications. Their impact has been particularly pronounced in photovoltaics, where power conversion efficiency (PCE) has risen dramatically from 3.8% in 2009 [1] to over 27% today [2, 3]. This success has further spurred breakthroughs in other areas, including photodetectors (PDs) [4, 5], lasers [6, 7], light‐emitting diodes (LEDs) [8, 9, 10], radiation detectors [4, 11] and thin‐film transistors (TFTs) [12, 13, 14, 15, 16].

OMHP adopts the general formula ABX_3_, where A is a monovalent cation (e. g., cesium (Cs^+^), methylammonium (MA^+^), or formamidinium (FA^+^)), B is a divalent metal (e. g., lead (Pb^2+^) or tin (Sn^2+^)), and X is a halide ion (chlorine (Cl^−^), bromine (Br^−^), or iodine (I^−^) [17]. Notably, the incorporation of both Pb and Sn as divalent cations narrows the bandgap beyond the range attainable by purely Pb‐based perovskites [18], enabling applications in all‐perovskite tandem solar cells [19], near‐infrared PDs [20], and LEDs [21]. Despite these advances, the fundamental charge transport physics of such Sn‐Pb hybrid perovskites remains poorly understood.

TFTs offer a reliable platform to probe long‐range charge transport in semiconductors beyond the localized information provided by spectroscopic techniques, which typically yield only upper limits on mobility [22]. The first perovskite TFT, reported in 1999 [23], employed a 2D perovskite and achieved a mobility of 0.6 cm^2^V^−1^s^−1^. Subsequent research focused on enhancing the performance of 2D and mixed‐dimensional Ruddlesden–Popper (R–P) perovskites through processing strategies and device engineering [24]. More recently, 3D Sn‐based perovskite TFTs have demonstrated exceptional mobilities approaching 50 cm^2^ V^−1^ s^−1^, but their practical implementation has been severely hampered by poor stability due to the easy oxidation of Sn^2+^ to Sn^4+^ [25]. By contrast, engineering the A and X sites in 3D Pb‐based perovskites has revealed to significantly influence charge transport in TFTs [26, 27, 28]. Nevertheless, issues such as undesired ion migration and associated instabilities remain pervasive, especially under ambient conditions [29, 30, 31]. These challenges underscore the urgent need for effective strategies to simultaneously enhance the mobility and suppress defect‐mediated degradation in Sn‐Pb mixed perovskites.

In this regard, recent studies on Sn–Pb perovskite FETs have highlighted the potential of compositional engineering, additive‐assisted crystallization, and interface passivation to achieve both high mobility and operational stability. Benchmark demonstrations have reported hole mobilities exceeding 70 cm^2^V^−1^s^−1^ through triple‐cation alloying and film‐quality optimization [32], while 2D/3D heterostructures and molecular passivation layers have been shown to mitigate interfacial trap states and bias stress effects [33]. Vapor‐deposition approaches and controlled surface oxidation strategies have further enabled enhancement‐mode operation and reduced gate leakage [34]. Despite these advances, maintaining long‐term stability under ambient conditions remains the primary bottleneck, motivating continued exploration of additive engineering as a practical pathway toward robust Sn–Pb TFTs [35].

Here, we propose a simple yet powerful additive engineering approach by incorporating the molecule 4,8‐Dihydrobenzo[1,2‐b:4,5‐b']dithiophen‐4,8‐dione (BDTD) into the MA_0.4_FA_0.6_Sn_0.5_Pb_0.5_I_3_ precursor. BDTD coordinates with undercoordinated Sn and Pb atoms via its sulfur and oxygen groups, thereby alleviating microstrain, reducing defect density, and promoting larger grain growth. As confirmed by atomic force microscopy (AFM) and scanning electron microscopy (SEM) analyses, BDTD‐treated films exhibit enhanced crystallinity and smoother morphology, resulting in an order‐of‐magnitude increase in hole mobility (4.1 vs. 0.38 cm^2^ V^−1^ s^−1^) and significant suppression of hysteresis. These results establish additive engineering as a promising route to overcome the intrinsic limitations in Sn‐Pb perovskite TFTs, paving the way toward stable, high‐performance complementary circuits

## 2 Results and Discussion

**FIGURE 1:**
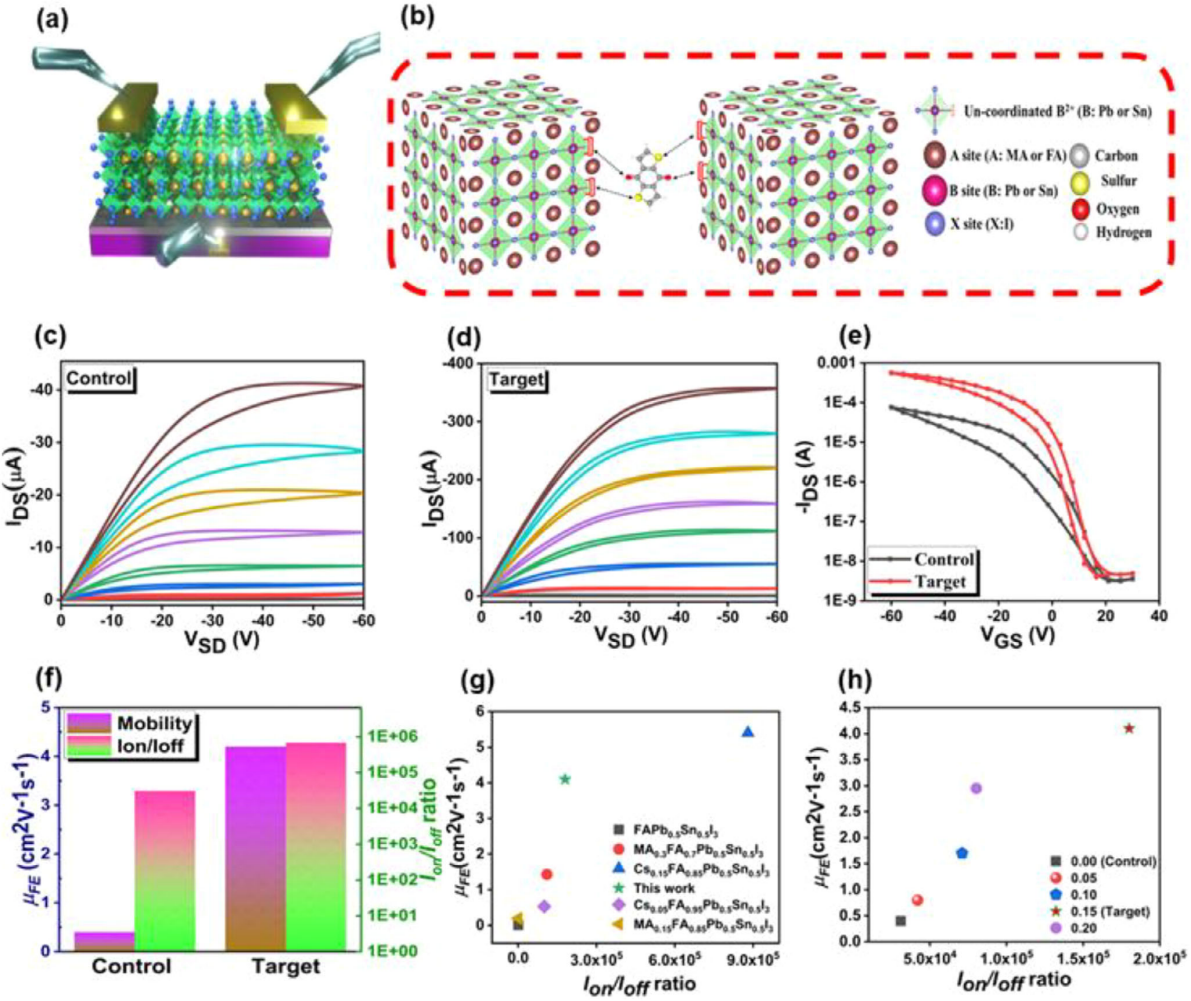
Device architecture and electrical performance of Sn–Pb perovskite TFTs with and without BDTD. (a) Schematic of the BGTC configuration. (b) Illustration of BDTT interaction with undercoordinated Sn^2+^/Pb^2+^ ions, illustrating defect passivation and microstrain relief. (c,d) Output characteristics of control and BDTD‐treated devices with gate voltage ranging from +10 V to –60 V with a step of –10 V at 300 K. (e) Transfer curves (*L* = 70 µm, *W* = 1700 µm) at *V_DS_* = −40 V, exhibiting p‐type behaviour. (f) Extracted *µ_h_* and *I_on_/I_off_* ratio for both devices. (g) Comparison of *µ_FE_* and *I_on_/I_off_* values with previously reported spin‐coated perovskite TFTs. (h) Dependence of *µ_FE_* and *I_on_/I_off_* on BDTD concentration, highlighting 0.15 mg as the optimized condition.

Figure 1a shows the architecture of the bottom‐gate, top‐contact (BGTC) TFT, whose active channel consists of a Sn‐Pb‐based perovskite layer fabricated with or without the BDTD additive. Gold (Au) electrodes define the source (S) and drain (D), SiO_2_ serves as the gate dielectric, and heavily doped Si functions as the gate (G). A schematic illustration of the molecular interaction in Figure 1b highlights the coordination of oxygen atoms from the dione group and sulfur atoms from the thiophene group in BDTD with undercoordinated Sn^2+^ and Pb^2+^ ions.

The output characteristics of the control and BDTD‐modified devices are displayed in Figure 1c,d, respectively. The BDTD‐treated transistor shows a remarkably improved performance, exhibiting more linear behavior at low drain–source voltages (VDS) and clear current saturation at higher VDS. Notably, the maximum drain current (IDS) increases by nearly an order of magnitude—rising from 40 µA in the control device to 360 µA in the BDTD‐treated device. The device features a channel length of 70 µm and a channel width of 1,700 µm, with these dimensions confirmed by scanning electron microscopy (SEM), as shown in the supporting data (Figure S1a–b). This result confirms reduced injection barrier and improved ohmic contact between the perovskite channel and Au electrodes. Figure 1e presents the p‐type transfer characteristics at *V_DS_* = −40 V. From these data, the field‐effect hole mobility (*µ_h_*) and on/off current ratio (*I_on_/I_off_*) were extracted. As shown in Figure 1f, The optimized 0.15 mg BDTD device delivered a maximum (average) µ_h_ of 4.1 (3.95 ± 0.12) cm^2^ V^−1^ s^−1^ and an Ion/Ioff ratio of (1.8 × 10^5^ ± 2.06 × 10^5^), whereas the control device showed only 0.38 (0.35 ± 0.05) cm^2^ V^−1^ s^−1^ and (3.1 × 10^4^ ± 2.37 × 10^4^), respectively. Supporting data in Figures S2 and S3 and Table S1 confirm the strong dependence of electrical performance on additive concentration: both *µ_h_* and *I_on_/I_off_* ratio steadily increased as BDTD loading rose from 0 to 0.15 mg; but both parameters exhibited a declining trend when the addition was further increased to 0.2 mg, likely due to the insulating effect caused by excess BDTD. The corresponding increase in threshold voltage (*Vth*) supports this interpretation. Compared with previously reported Sn‐Pb perovskite TFT benchmarks, the obtained field‐effect mobility (*µ_FE_*) and *I_on_/I_off_* ratios are among one of the highest ever reported for spin‐coated Sn‐Pb perovskite TFTs, surpassing the performance of most devices using similar material systems, as summarized in Figure 1g and Table S2. Figure 1h also illustrates the trends of *µ_FE_* and *I_on_/I_off_* as a function of different BDTD concentrations.

**FIGURE 2:**
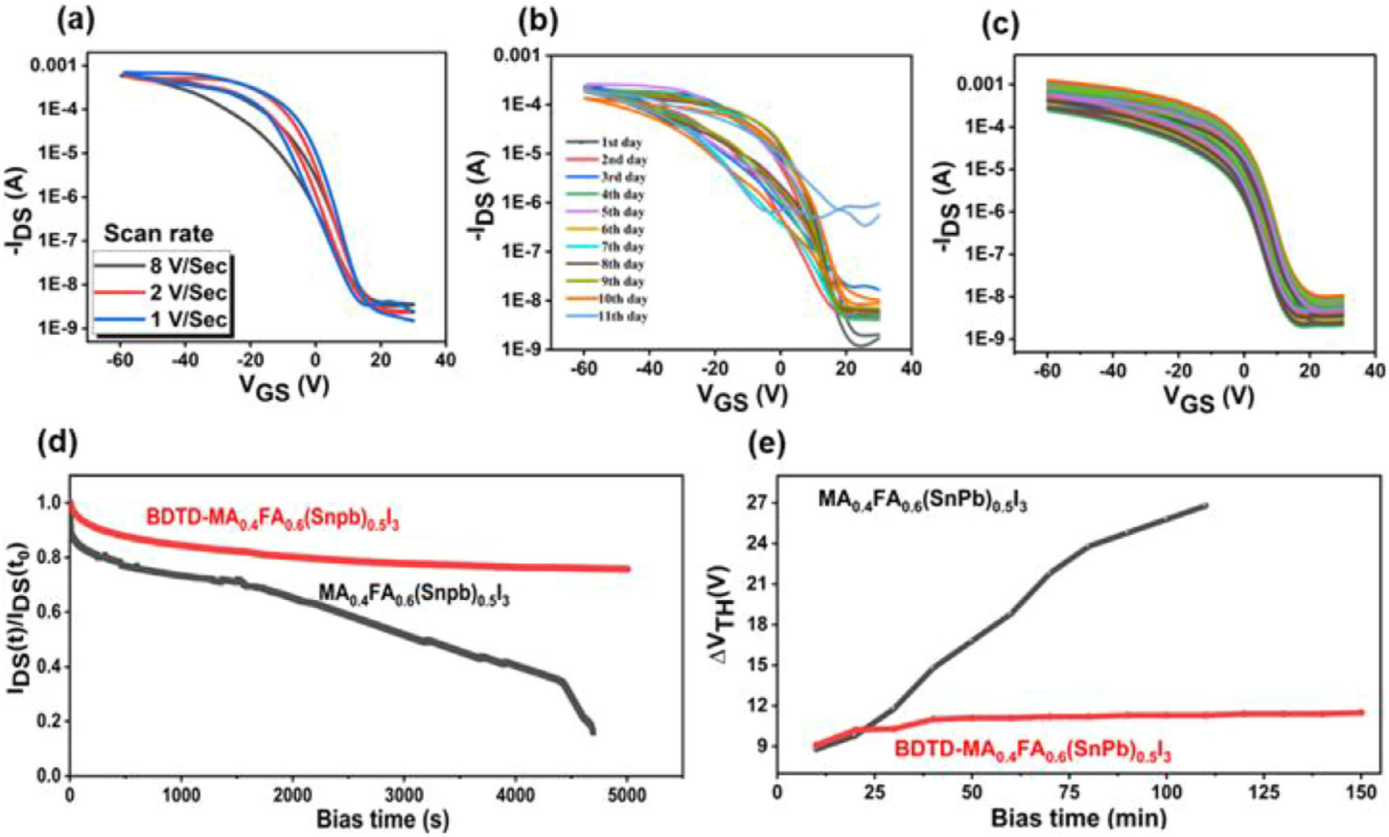
Stability and reproducibility of optimized Sn–Pb perovskite TFTs incorporating BDTD. (a) Transfer characteristics recorded at different scan rates (1, 2, and 8 V s^−1^) at 300 K, confirming robust operation. (b) Long‐term stability test over 11 days, showing negligible channel current decay and minimal *ΔV_th_* (≈ 10–11.8 V). (c) Device reproducibility: Transfer curves from 40 transistors across eight independent batches, yielding consistent *µ_FE_* and *I_on_/I_off_*. (d) Bias‐stress reliability testing (*V_GS_* = −60 V and *V_DS_* = −40 V). The BDTD‐treated device shows superior *I_DS_* retention. (e) Comparison of *ΔV_th_* under continuous stress between control and BDTD‐modified devices, highlighting enhanced operational stability.

To evaluate device stability, transfer characteristics of Sn‐Pb‐based TFTs with the BDTD additive were measured at 300 K with different scan rates, as shown in Figure 2a. The results demonstrate stable operation across the entire scan rate range, confirming enhanced operational robustness. Figure 2b displays transfer curves for a ten‐day long‐term monitoring test, further revealing excellent device performance persistence under N_2_‐filled glovebox (O_2_< 0.1 ppm, H_2_O < 0.1 ppm). With channel currents spanning five orders of magnitude, the threshold voltage shift (*ΔV_th_*) exhibited only minor variations between ≈10 and 11.8 V, underscoring the optimized chemical and structural stability of the film. Reproducibility was also confirmed: Data from 40 devices across eight independent batches yielded an average *µ_FE_* of 4.1 cm^2^·V^−1^·s^−1^ and an *I_on_/I_off_* ratio exceeding 10^5^, as summarized in Figure 2c. Long‐term bias stressing testing was conducted under an inert nitrogen atmosphere with constant negative gate and drain voltages (*V_GS_* = −60 V, *V_DS_* = −40 V). As depicted in Figure 2d,e, the optimized MA_0_._4_FA_0_._6_(PbSn)_0_._5_I_3_ FET device demonstrated outstanding reliability: after continuous operation exceeding 5000 s, *ΔV_th_* remained below 1.5 V, and the *I_DS_* decay was less than 15%. Such performance significantly suppresses most reported Sn‐based perovskite TFTs, highlighting the synergistic role of BDTD in suppressing defect‐induced instability and promoting stable charge transport. In sharp contrast to BDTD‐ modified devices, transistors fabricated without the BDTD additive exhibited rapid degradation under bias stress, as shown in Figure S4b. Its *V_TH_* shifted significantly by approximately 24 V, accompanied by a steep decay in *I_DS_* within 4000 s. In contrast, the BDTD‐treated device maintained stable current output with negligible *V_th_* drift (as shown in Figure S4a), confirming that device degradation is strongly influenced by additive‐mediated film quality.

**FIGURE 3:**
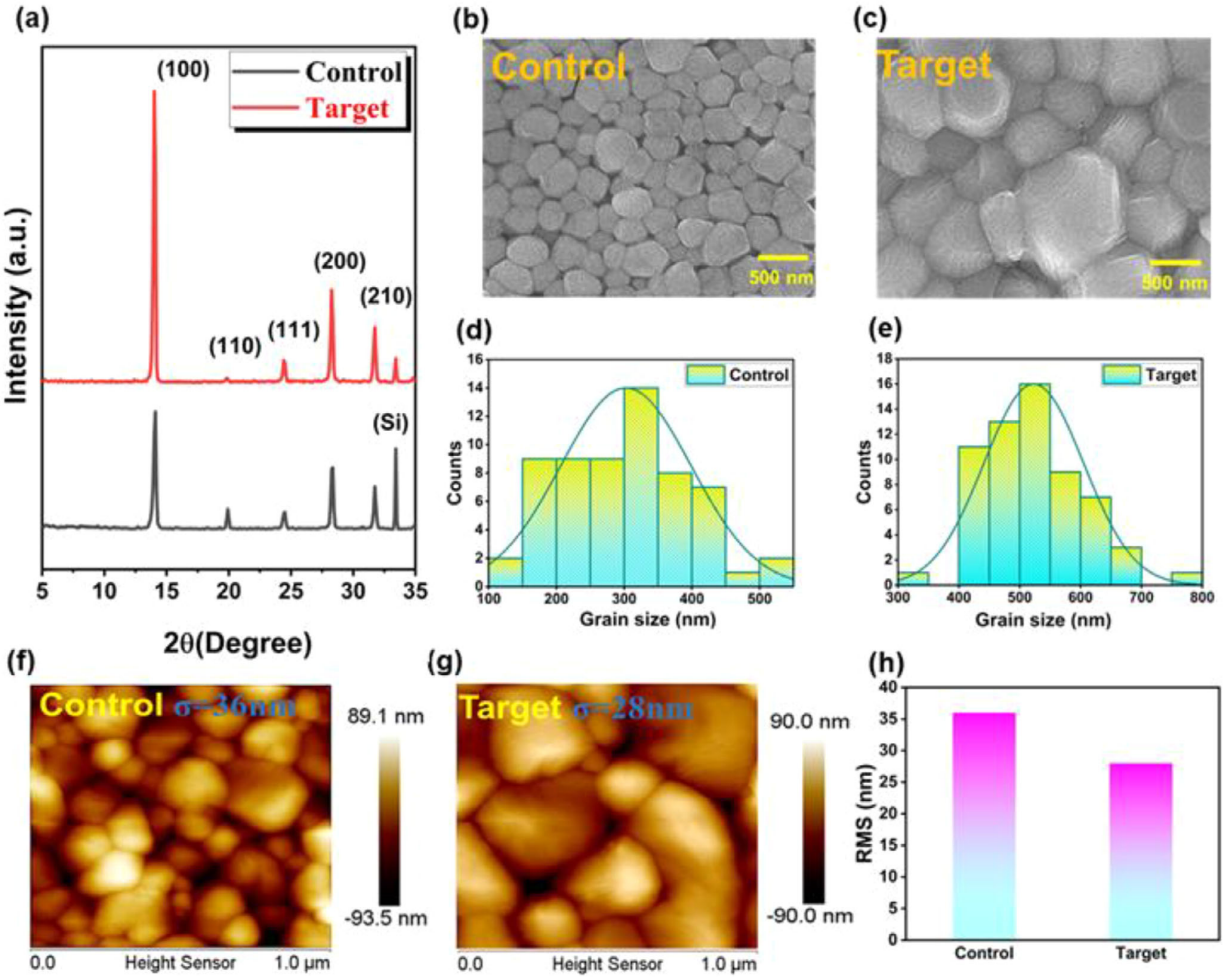
Structural and morphological analysis of Sn‐Pb perovskite films with and without BDTD. (a) XRD patterns showing enhanced peak intensity and reduced FWHM with additive incorporation. (b,c) SEM images of control and BDTD‐treated films. (d,e) Grain size distributions revealing increased grain size and densification at the optimized additive concentration. (f,g) AFM topography images, highlighting reduced surface roughness in BDTD‐modified films.

The effect of BDTD on crystallization was first examined by X‐ray diffraction (XRD) analysis. As shown in Figure 3a, both the control (without BDTA) and the target (with 0.15 mg/mL BDTA) films exhibit reflection peaks at 14°, 24.5°, 28.25°, and 31.7°, corresponding to the (100), (111), (200), and (210) lattice planes, consistent with a cubic perovskite structure [28]. Figure S5 shows XRD patterns of Sn‐Pb perovskite films prepared with varying BDTD concentrations. Importantly, the target film exhibits significantly enhanced peak intensities and narrower full‐width at half maximum (FWHM) values in Figure S6, indicating enhanced crystallinity.

The ultraviolet–visible (UV–vis) absorption spectra and Tauc plot analysis of MA_0.4_FA_0.6_Sn_0.5_Pb_0.5_I_3_ films with varying BDTD concentrations were recorded, with results presented in Figure S7a–c. The results reveal indicate that BDTD has a negligible effect on the bandgap (the control film: *E_g_* = 1.22 eV; the target films: 1.23 eV), suggesting that additive incorporation modulates film quality without perturbing its intrinsic electronic structure. Stability testing further highlights BDTD's benefits: as shown in Figures S8 and S9, XRD and UV–vis analyses of aged samples reveal lattice distortion, peak broadening, and reduced absorption intensity in the untreated film, consistent with defect formation and Sn^2+^ oxidation. By contrast, BDTD‐containing film preserve crystallinity, sharp absorption onset, and stable diffraction peaks, demonstrating their ability to suppress ion migration and enhance structural integrity over time [37, 38]. To further assess the impact of BDTD on the crystallization behavior of perovskite films, scanning electron microscopy (SEM) analysis was conducted. Figure 3b–c displays the surface morphology of the control and target films. The control film shows relatively small grains (440–455 nm), with an non‐uniform and loosely stacked surface structure. This poor compactness is often associated with a rapid, uncontrolled crystallization process, potentially leading to a high density of grain boundaries and defects. In contrast, the target film formed larger grains (600–610 nm) with densely stacked grains [29]. The improved morphology arises from Lewis acid–base coordination between electron‐rich atoms in BDTD (Lewis base) and undercoordinated Sn^2+^/Pb^2+^ ions (Lewis acids) [30], which delays nucleation and promotes more orderly crystal growth. Figure S10 provides additional SEM images of films prepared with varying BDTD concentrations, with corresponding grain size data summarized in Table S3. The grain size statistics in Figure 3d–e confirm an increase in average grain size from 442 nm (control) to 602 nm (target). This increase in grain size, along with improved film uniformity and compactness, plays a critical role in device performance. Larger grains reduce the number of grain boundaries — areas that are common sites for charge traps thereby enhancing charge transport and reducing recombination losses within the perovskite layer.

Atomic force microscopy (AFM) further elucidates the effects of additive engineering, as shown in Figures 3f–g and S11a–e. The MA_0.4_FA_0.6_Sn_0.5_Pb_0.5_I_3_ films were deposited onto Si/SiO_2_ substrates. The control films exhibited small grain sizes and poor surface coverage, consistent with previously reported results [31]. This is primarily attributed to the rapid crystallization of Sn‐Pb perovskites, which often leads to suboptimal film morphology characterized by irregular grain distribution and numerous pinhole defects. Such defects form charge traps and leakage current pathways, severely impairing device performance. Conversely, adding BDTD into the perovskite precursor significantly improved film quality. The target film surface becomes smoother and more uniform, with a marked reduction in pinholes. As the BDTD concentration increases from 0.05 to 0.15 mg/mL, surface roughness exhibits a clear decreasing trend. Specifically, the root‐mean‐square (RMS) roughness decreased from 36 nm for the control film to 27 nm for the film containing 0.15 mg/mL BDTD, indicating a much smoother surface. This reduction in surface roughness is critical for TFTs, because smoother perovskite films provide better physical contact with electrodes, thereby facilitating more efficient charge injection and extraction at the interface. The RMS values for all studied films are summarized in Figure S11f. However, when the BDTD concentration exceeded 0.15 mg/mL, the RMS roughness exhibited a slight increase. This is likely due to excessive heterogeneous nucleation in the precursor solution, a process that may introduce new surface irregularities and compromise film uniformity. Collectively, these results emphasize BDTD's effective modulation of perovskite crystallization, optimizing grain size and surface morphology. By controlling these parameters, BDTD enhances the structural quality of perovskite films, which is expected to improve device electronic performance.

Based on AFM results, a BDTD concentration of 0.15 mg/mL was identified as the optimal condition for achieving the smoothest film morphology. This optimized formulation was subsequently characterized via grazing incidence wide‐angle X‐ray scattering (GIWAXS) to investigate its influence on the crystal structure of MA_0.4_FA_0.6_Sn_0.5_Pb_0.5_I_3_ films. As presented in Figure S12a–b [36], the control film exhibits nearly isotropic diffraction rings, reflecting random orientation of crystalline domains and a lack of long‐range order within the film. In contrast, the target film displays a pronounced preferred orientation along the (100) plane, indicating more regular and directional crystal growth. This implies that the additive facilitates better alignment of perovskite crystals, potentially achieved by influencing nucleation and growth kinetics during film formation. Moreover, asymmetric diffraction peaks observed in the control film (Figure S13) showed extended tails — an indication of phase impurities or inhomogeneities, possibly originating from uneven crystallization or additive‐deficient regions. These elongated tails may also suggest minor phase segregation or poorly integrated domains. In contrast, the optimized film displayed sharp, symmetric, and narrower diffraction peaks, indicating enhanced overall crystallinity and improved uniformity. Such well‐defined crystal features are highly desirable for electronic applications, as they reduce structural defects and grain boundary‐related trap states, thereby enabling efficient charge transport by. Overall, these GIWAXS results further validate the role of BDTD in directing the crystallization process and improving the structural order of Sn‐Pb perovskite films.

**FIGURE 4:**
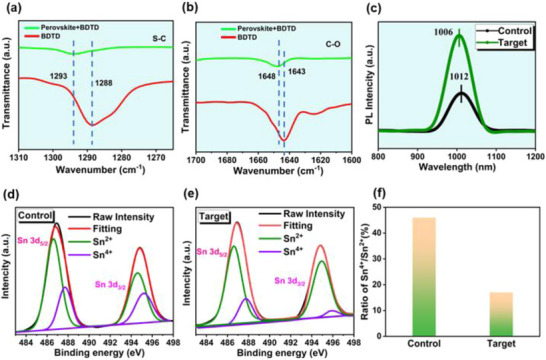
Molecular interactions and defect passivation induced by BDTD. (a, b) FTIR spectra showing C–S and C = O vibrational shifts upon coordination with the perovskite lattice. (c) PL spectra demonstrating enhanced radiative recombination in BDTD‐treated films. (d,e) XPS spectra of Sn 3d core levels for control and BDTD‐containing films. (f) Comparison of Sn^4+^/Sn^2+^ ratios, confirming suppressed oxidation in additive‐modified samples.

To investigate the chemical interactions between the BDTD additive and the perovskite precursor, Fourier Transform Infrared spectroscopy (FTIR) analysis was conducted, with results shown in Figures 4 and S14. In Figure 4a, the characteristic C–S stretching vibration originally located at 1288 cm^−1^, shifted to 1293 cm^−1^ after adding BDTD into the perovskite system. This shift suggests the formation of hydrogen bonds between sulfur atoms in BDTD and organic cations, such as MA^+^ (methylammonium) or FA^+^ (formamidinium) [37]. Similarly, Figure 4b highlights changes in the C = O stretching vibration. In pure BDTD, this vibrational peak appears at 1643 cm^−1^, but shifts to 1648 cm^−1^ upon interaction with the perovskite precursor. This upshift indicates strong coordination interactions between the carbonyl oxygen atoms of BDTD and the undercoordinated Sn^2+^ or Pb^2+^ ions within the perovskite structure. Such interactions are likely driven by hydrogen bonding or Lewis acid–base interactions, contributing to the stabilization of the perovskite lattice and reduction of defect states [38]. These FTIR results unequivocally confirm that BDTD not only interacts with the organic components of the perovskite but also plays a significant role in bonding with metal cations, thereby improving structural stability and potentially influencing crystallization processes.

To evaluate the impact of Sn^2+^ oxidation on charge carrier dynamics, steady‐state photoluminescence (PL) measurements were carried out on mixed Sn‐Pb perovskite films, as illustrated in Figures 4c and S15. Compared to the control film, the optimized perovskite films exhibit stronger PL intensity. This enhancement indicates a reduction in non‐radiative recombination losses or an increase in radiative recombination efficiency. Such behavior is typically associated with a decrease in defect states within the perovskite film, as these defects trap carriers and facilitate non‐radiative pathways [39].

In mixed Sn‐Pb perovskites, the oxidation of Sn^2+^ to Sn^4+^ poses a significant challenge, potentially compromising both device performance and long‐term stability. To evaluate the ability of BDTD to suppress this undesirable oxidation, X‐ray photoelectron spectroscopy (XPS) was employed for characterization. Figure 4d–e presents the Sn 3d_3_/_2_ and Sn 3d_5_/_2_ core‐level spectra of MA_0.4_FA_0.6_Sn_0.5_Pb_0.5_I_3_ films, where (d) shows the control film without BDTD addition, and (e) displays the target film containing the additive. In the control sample, Sn^2+^ signals appeared at binding energies of 486.60 eV (3d_5_/_2_) and 495.0 eV (3d_3_/_2_), while the oxidized Sn^4+^ appeared at 487.5 and 495.8 eV, respectively. For the BDTD‐incorporated film, the Sn^2+^ peaks remained at similar positions (486.60 and 495.1 eV), but the relative intensities of the Sn^4+^ peaks (487.4  and 495.8 eV) were significantly reduced, as detailed in Table S4. This attenuation indicates a decrease in Sn^4+^ concentration within the target film, demonstrating that the additive effectively stabilizes the Sn^2+^ oxidation state. As summarized in Figure 4f, the Sn^4+^/Sn^2+^ ratio in the BDTD‐containing film is markedly lower than in the control group. This improvement stems from BDTD's electron‐donating properties, which increase the electron density around Sn^2+^ ions, thereby reducing their oxidation susceptibility. These findings confirm BDTD's pivotal role in inhibiting Sn^2+^ oxidation, ultimately enhancing chemical stability and device reliability.

**FIGURE 5:**
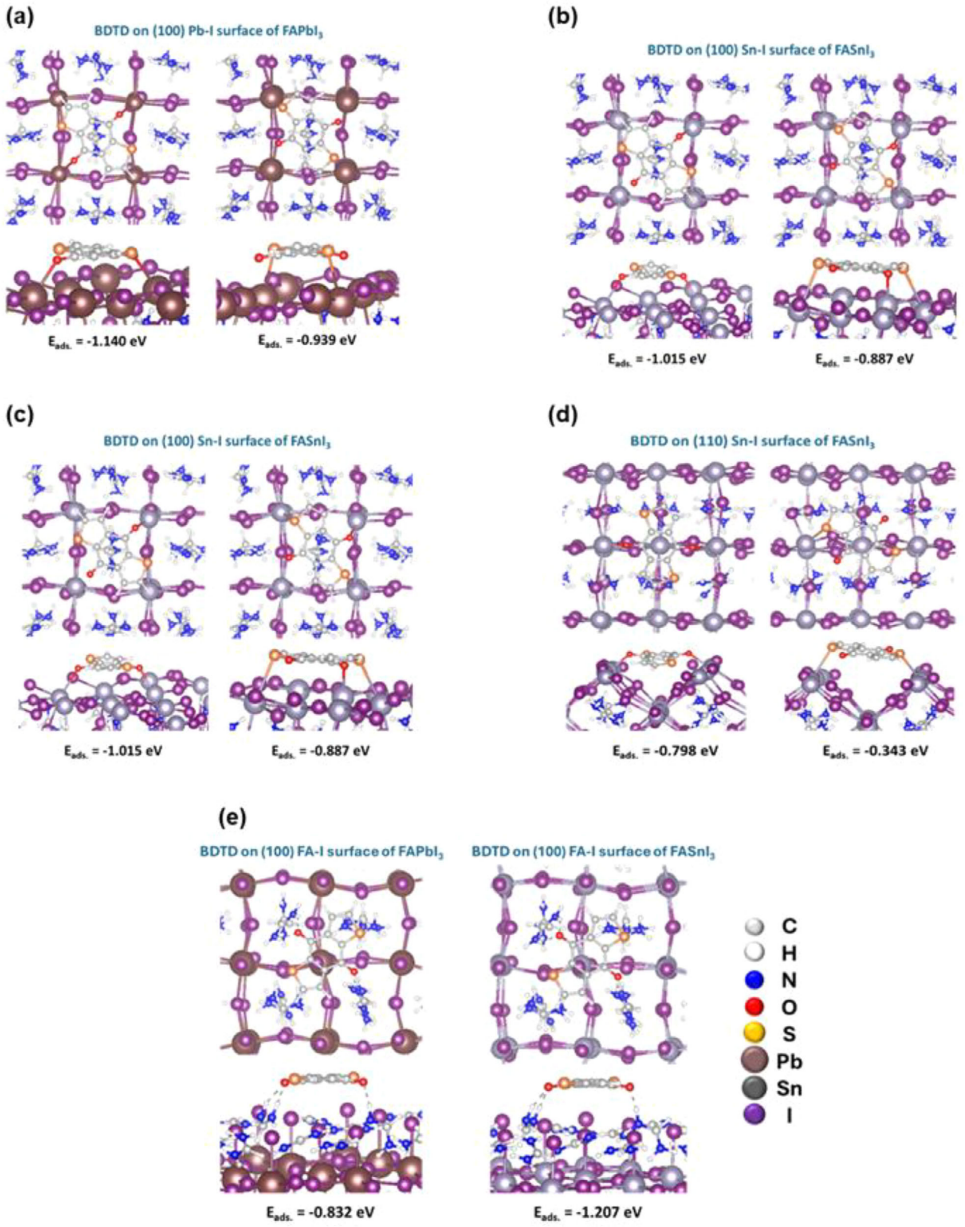
Density functional theory: Geometry‐optimized structures and adsorption energies of BDTD on (a,b) FAMI_3_ (100) M‐I terminated surfaces, (c,d) FAMI_3_ (110) M‐I terminated surfaces, and (e) FAMI_3_ (100) FA‐I terminated surfaces.

To investigate the interaction between BDTD and the FAMI_3_ perovskite surfaces (where M = Pb or Sn), density functional theory (DFT) calculations were performed. As illustrated in Figure S17, multiple exposed facets and terminated surfaces of FAMI_3_ were modelled to investigate the adsorption affinity of BDTD. Geometric optimization was performed after placing a single BDTD molecule on each surface configuration. As shown in Figure 5, BDTD exhibits strong interactions with perovskite surfaces, particularly pronounced on M–I terminated surfaces. This behavior is attributed to the strong Coulombic interactions between perovskite and BDTD, wherein both oxygen and sulfur atoms in BDTD effectively coordinate with metal cations. Among the examined configurations, the Pb–I terminated (110) surface of FAPbI_3_ demonstrates the highest adsorption affinity for BDTD. Moreover, the interaction between FAPbI_3_ and BDTD tends to be stronger than that with FASnI_3_, consistent with the ESP‐derived atomic charge distribution analysis: as illustrated in Figure S17a, lead atoms exhibit higher positive charges than tin atoms. Additionally, the FA–I terminated (100) surface of FAMI_3_ also promotes BDTD adsorption, primarily through hydrogen bonding between the oxygen atom in BDTD and the amine group of FA. In contrast, the FA–I terminated (110) surface of FAMI_3_ displays the weakest interaction with BDTD. This is due to the strong negative charge of iodine (Figure S17b), which induces significant Coulombic repulsion between the oxygen and sulfur atoms in BDTD.

To advance the performance of perovskite‐based transistors, particularly in optimizing *µ_h_* and *V_th_*, we adopted a surface passivation approach using tetrabutylammonium hexafluorophosphate (TBAPF_6_) [40]. This compound was introduced to address surface defects in the perovskite layer. As shown in Figure S18, TBAPF_6_ uniformly coats the perovskite surface, effectively passivating undercoordinated Pb^2+^ sites known to form charge‐trapping states that hinder charge transport. Moreover, the long hydrophobic alkyl chains in TBAPF_6_ form a physical barrier that helps shield the perovskite film from moisture‐related degradation a common stability issue in perovskite devices [41]. Due to this dual effect (chemical passivation and physical protection), Figure S19 shows improvements in both *µ_h_* and *V_th_*. Notably, the *V_th_* decreased significantly from 10.1 to 3.6 V with increasing TBAPF_6_ concentration, reaching an optimum at 3 mg/mL. However, beyond this concentration, *V_th_* slightly increased, suggesting a threshold beyond which excess additive may disrupt the film morphology or induce new defects. Table S5 summarizes the *V_th_*, *µ_h_*, and *I_on_/I_off_* ratios at different concentrations. These results clearly demonstrate that at the optimal concentration, TBAPF_6_ significantly enhances key transistor parameters by minimizing defect‐induced non‐radiative recombination and improving charge transport. Nonetheless, to further refine this strategy, a deeper understanding of the interaction mechanisms between TBAPF_6_ and the perovskite surface remains necessary.

**FIGURE 6:**
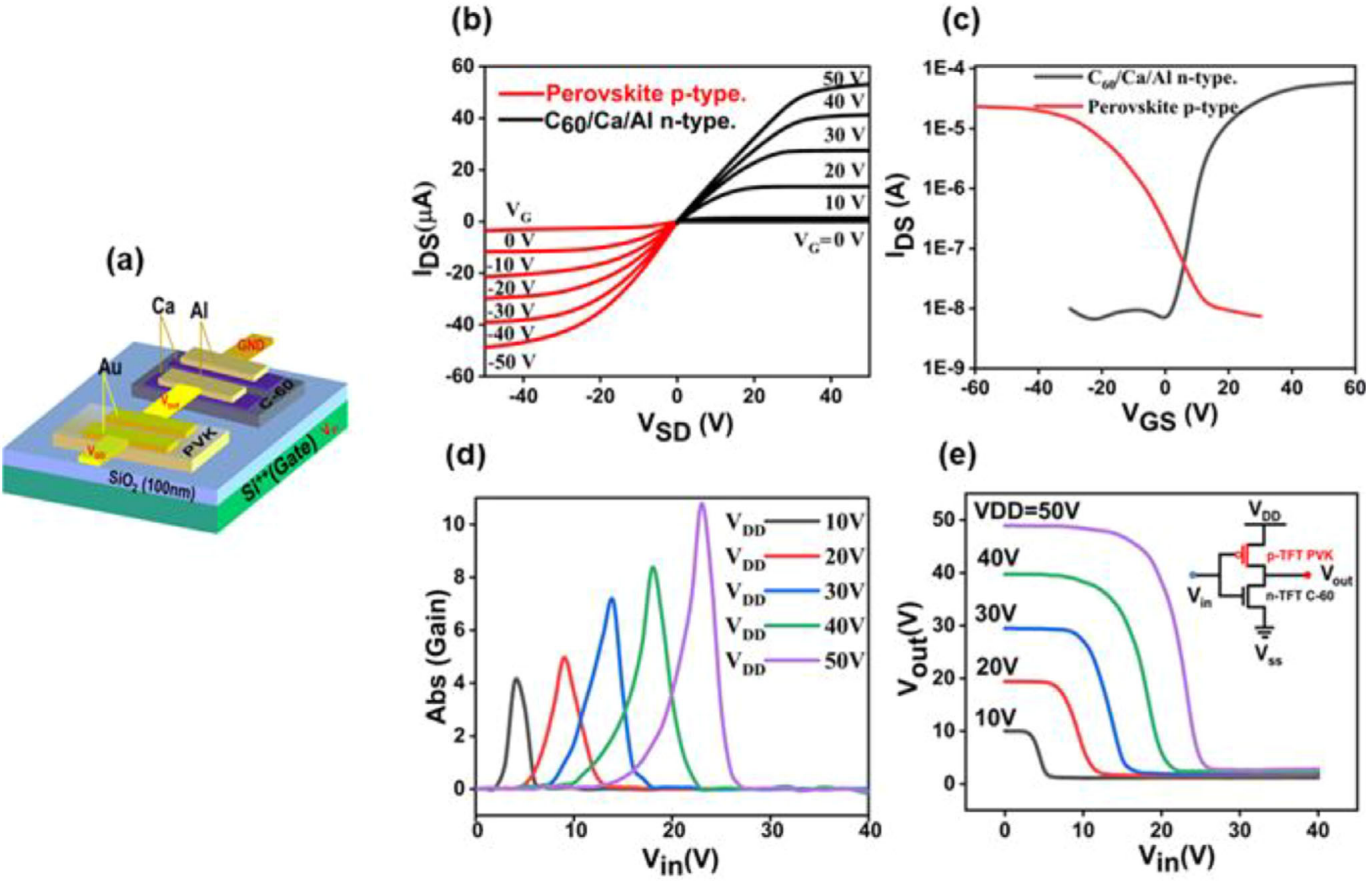
(a) Schematic of a complementary TFT structure using SiO_2_ as the gate dielectric layer. (b) Output characteristics of perovskite and C_60_‐based TFTs. (c) Transfer characteristics measured at *V_D_* = ±40 V. (d) Voltage transfer characteristics (VTCs) of the inverter at different *V_DD_* values; inset shows the inverter circuit structure. (e) Voltage gain of the inverter as a function of varying V_DD_.

To validate the practical potential of optimized device, a complementary inverter circuit was fabricated by integrating a p‐type MA_0.4_FA_0.6_Sn_0.5_Pb_0.5_I_3_ TFT with an n‐type C_60_ transistor. Figure 6a,d present the circuit layout and equivalent circuit, respectively, while the single‐transistor characteristics of the perovskite‐based p‐type and C_60_‐based n‐type devices are presented in Figure 6b,c. Both devices display balanced and well‐defined output/transfer characteristics, validating their suitability for complementary integration. Figure 6e presents the voltage transfer characteristics (VTCs) of the complementary inverter under different supply voltages (VDD = 50, 40, 30, 20, and 10 V). The inverter demonstrates the expected logic behavior: when the input voltage (*V_in_*) is low (logic “0”), the output voltage (*V_out_*) is high (logic “1”), and vice versa. This reflects a typical inverter function, where the output state is always opposite to the input. Importantly, the inverter achieves full rail‐to‐rail switching, covers the entire VDD range with its output voltage, and exhibits a high voltage gain (defined as *dV_out_*/*dV_in_*), exceeding 10 at VDD = 50 V, as shown in Figure 6f. These results confirm that the mixed Sn‐Pb perovskite can serve as a robust p‐type material for complementary logic circuits, opening new possibilities for low‐cost, solution‐processed electronic devices.

## 3 Conclusion

In summary, we demonstrate that additive engineering with BDTD is a powerful strategy to achieve high‐performance MA_0.4_FA_0.6_Sn_0.5_Pb_0.5_I_3_ perovskite TFTs. BDTD regulates nucleation and crystal growth while suppressing Sn ^2+^oxidation through its Lewis base functionality and interaction with undercoordinated Sn^2+^/Pb^2+^ ions. The optimized film exhibits enhanced crystallinity, larger grain size, smoother surface, and lower defect density. Consequently, the transistor mobility increased by an order of magnitude (4.1 cm^2^ V^−1^ s^−1^ vs. 0.38 cm^2^ V^−1^ s^−1^ for the control device), exhibiting a superior *I_on_/I_off_* ratio (1.8 × 10^5^) alongside reduced hysteresis and excellent stability. The complementary strategy of using TBAPF_6_ as the surface passivator further enhances threshold voltage and device reliability. The integrated inverter circuit validates Sn–Pb perovskites as a viable p‐type semiconductor in CMOS‐like electronic devices. This study establishes additive engineering as a versatile approach to overcoming the intrinsic instability of Sn–Pb perovskites, advancing their application in next‐generation, solution‐processed complementary circuits.

## Author Contributions

Z.A.A. and C.W.C. conceived the project. C.W.C. directed the project. Z.A.A. and characterized the thin‐film transistor (TFT) devices. A.K. Performed XPS characterization. S.B. Performed FTIR characterization. Y.C.C., S.L., A.T., and C.H. formal analysis and data curation. P.Y.Y. and C.W.P. performed the DFT calculations and analysis. All authors participated in the preparation of the manuscript and commented on its content.

## Conflicts of Interest

The authors declare no conflicts of interest.

## Supporting information




**Supporting File**: advs73429‐sup‐0001‐SuppMat.docx.

## Data Availability

The data that support the findings of this study are available from the corresponding author upon reasonable request.
