# Supplementary material for: Synergistic Effects of Additive Engineering in Enhancing the Performance of Sn–Pb Perovskite Thin‐Film Transistors and Derived Logic Circuits
Source: Adv Sci (Weinh). 2026 Feb 4;13(13):e20241. doi: 10.1002/advs.202520241 (PMC12955910; doi:10.1002/advs.202520241)
Supplement: Supplementary file 1 — Supporting File: advs73429‐sup‐0001‐SuppMat.docx. [file ADVS-13-e20241-s001.docx]

Supporting Information

**Synergistic Effects of Additive Engineering in Enhancing the Performance of Sn–Pb Perovskite Thin-Film Transistors and Derived Logic Circuits**

*Zeeshan Alam Ansari, Abhishek Kumar, Soumallya Banerjee, Chintam Hanmandlu, Anjali Thakran, Yu-Te Chen, Po-Yu Yang, Shenghan Li, Chun-Wei Pao, Yun-Chorng Chang, Chu-Chen Chueh,* *Chih-Wei Chu^*^*

Z. A. Ansari, Y. C. Chang

Department of Physics, National Taiwan University, Sec. 4, Roosevelt Road, Taipei 106, Taiwan, Taiwan ROC

Z. A. Ansari, A. Kumar, S. Banerjee, C. Hanmandlu, A. Thakran, Yu-Te Chen*,* P. Y. Yang, S. Li, C. W. Pao, Y. C. Chang, C. W. Chu.

Research Center for Applied Sciences, Academia Sinica, 128, Academia Road, Section 2, Nangang, Taipei 11529, Taiwan, ROC

E-mail: [gchu@gate.sinica.edu.tw](mailto:gchu@gate.sinica.edu.tw)

Z. A. Ansari

Nano-Science and Technology Program, Taiwan International Graduate Program, Academia Sinica, Taipei 106, Taiwan ROC

C. C. Chueh

Department of Chemical Engineering, National Taiwan University, Taipei 106319, Taiwan, ROC

C. W. Chu

Department of Photonics, National Yang Ming Chiao Tung University, Hsinchu City 30010, Taiwan ROC

**Keywords:** Mixed Sn-Pb perovskites; thin-film transistor, additive engineering; defect passivation; complementary logic circuits

**Experimental Section**

***Materials:*** All chemicals were Purchased at their designated purity levels and utilized without any additional purification. Methylammonium iodide (MAI) and formamidinium iodide (FAI) were sourced from Great Cell Solar. Lead iodide (PbI_2_, 99.999%), tin iodide (SnI_2_, 99.999%) and tin fluoride (SnF_2_) were obtained from Sigma-Aldrich. 4,8-Dihydrobenzo[1,2-b :4,5-b' ]dithiophen-4,8-dione (BDTD) were procured from Lumtech Taiwan. The solvents used in this study, including N,N-dimethylformamide (DMF, 99.8%), chlorobenzene (CB, 99.8%), and dimethyl sulfoxide (DMSO, >99.9%), were purchased from Sigma-Aldrich. All chemicals and solvents were stored in a nitrogen-filled glovebox with oxygen and moisture levels maintained below 0.1 ppm.

***Preparation of Perovskite Precursor Solution***

The MA₀._4_FA₀_.6_Pb₀.₅Sn₀.₅I₃ (0.6 M) precursor solution was prepared by dissolving MAI, FAI, PbI₂, and SnI₂ in a DMF:DMSO solvent mixture (3:1 ratio) and stirring the solution on a hot plate at 50 °C for 2 hours. The SnF₂ solution (6 mg/mL in DMF) was stirred overnight (12 hours) at room temperature. For the BDTD solution, BDTD (2 mg/mL in DMF) was stirred for 2 hours. The prepared SnF₂ solution and BDTD solution were then added to the MA₀._4_FA₀_.6_Pb₀.₅Sn₀.₅I₃ precursor to obtain the final solution, maintaining a concentration of 10 mol% SnF₂ relative to SnI₂ while varying the amount of BDTD. The mixed solution was stirred on a hot plate at 50 °C for 20 minutes and then allowed to cool for 10 minutes before use. All precursor solutions were prepared in 4 mL glass vials and filtered through a 0.22 µm polytetrafluoroethylene (PTFE) membrane before film fabrication.

***Device Fabrication***

We fabricated thin-film transistors (TFTs) using a bottom-gate, top-contact device structure inside an N₂-filled glovebox. Heavily doped silicon (Si) substrates with a 100 nm thermally grown SiO₂ layer served as both the gate electrode and the dielectric layer. The substrates were initially cleaned in an ultrasonic bath using acetone, deionized water, and isopropyl alcohol for 20 minutes each. After drying in an oven at 110 °C, they were treated with UV-ozone for 15 minutes to enhance surface wettability. The perovskite precursor solution was then spin-coated onto the SiO₂/Si substrates at 5,000 rpm for 25 seconds. After 15 seconds of spinning, 80 μL of chlorobenzene (CB) was carefully dropped onto the spinning substrate. This modified solution was spin-coated under the same conditions as the control perovskite solution and subsequently annealed at 100 °C for 10 minutes. The transistor fabrication was finalized by depositing Au source and drain electrodes (50 nm) via thermal evaporation under high vacuum (<10⁻⁶ Torr) using a shadow mask. The thermal evaporator was housed within an N₂-filled glovebox. The TFTs had a channel length of 70 μm and a channel width of 1,700 μm, channel dimensions were determined from scanning electron microscopy (SEM)


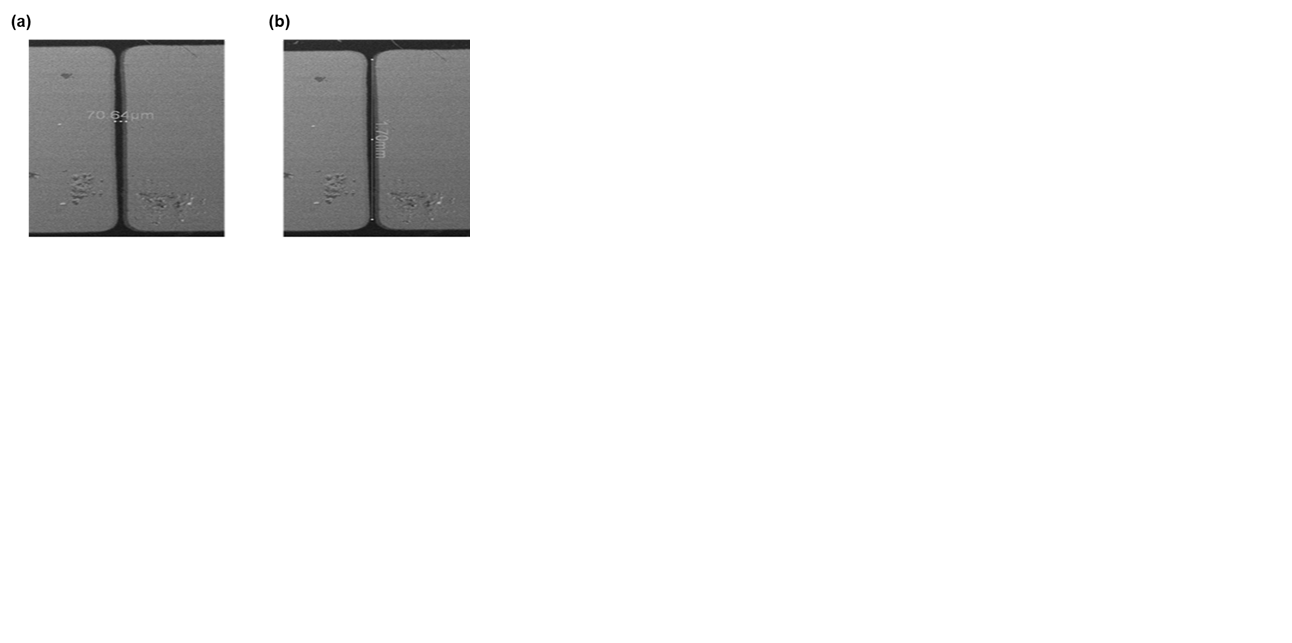


**Figure S1. (a)** Channel length and **(b)** width of fabricated devices

***Device characterization***

All the perovskite TFTs were characterized at room temperature in the dark inside an N₂-filled glovebox (O₂ < 0.1 ppm, H₂O < 0.1 ppm) using a Keysight/Agilent 4156C Precision Semiconductor Parameter Analyzer. The field-effect mobility (μₙ) of the TFTs was calculated in the saturation region from the forward transfer curves using the following equation.

$\mu\mathrm{FE}=\frac{2L}{WCi}\left( \frac{\delta\sqrt{IDS}}{\delta VGS} \right)$*_2_*

where *L*, *W* and *C*i are the channel length, channel width and areal capacitance of the dielectric respectively. Further, *I*_DS_ and *V*_GS_ are the source–drain current and gate–source voltage, respectively; *V_TH_* was estimated by linearly fitting (I*_DS_*)^0.5^ with respect to *V_GS_.*

Scanning electron microscope (SEM) images were obtained using a Quanta 200 FEG field-emission scanning electron microscope (FE-SEM, FEI Co.). Atomic force microscopy (AFM) images were captured with a Bruker Dimension Edge instrument. Steady-state photoluminescence (PL) measurements were performed using a photoluminescence spectrometer (FL900, Edinburgh Instruments, England). Fourier transform infrared (FTIR) spectra were recorded with a PerkinElmer FT-IR spectrometer. X-ray diffraction (XRD) patterns were acquired using a Bruker D8 Advance ECO X-ray diffractometer with Cu Kα radiation (λ = 1.5406 Å) over a 2θ range of 5–45° with a step size of 0.001°. X-ray photoelectron spectroscopy (XPS) measurements were carried out using a PHI 5000 Versa Probe equipped with an Al Kα X-ray source. XPS data fitting was done using a linear background and an asymmetric Gaussian-Lorentzian sum function. The UV-Visible/NIR absorption spectra of the perovskite films were measured with a JASCO V-770 UV-Visible/NIR spectrophotometer. The Space Charge Limited Current (SCLC) measurements were performed on devices with the structure ITO/PTAA/Perovskite (with and without BDTD)/Spiro-OMeTAD/Ag. The SCLC measurements were carried out at room temperature inside a glove box under dark conditions.

***Density Functional Theory***

All calculations were performed using periodic density functional theory (DFT), as implemented in the Vienna Ab-initio Simulation Package (VASP).^[1, 2]^ The all-electron projected augmented wave (PAW) pseudopotential.^[3]^ was employed to describe the core-valence interactions. The Perdew-Burke-Ernzerhof (PBE) exchange-correlation functional,^[4]^ within the generalized gradient approximation (GGA), was utilized. A kinetic energy cutoff was set to 500 eV. Electronic occupations were determined using the Gaussian smearing method, with a smearing width of 0.03 eV. To accurately account for van der Waals interactions, the Grimme dispersion correction (DFT-D3)^[5]^ was applied. The convergence criterion for electron relaxation using the self-consistent solution of the Kohn–Sham equations is fixed at 10^–6^ eV. For geometry optimizations, the convergence criterion was a maximum force on each atom of less than 0.05 eV/Å. The Brillouin zone was sampled using the Monkhorst–Pack method ^[6]^ with a 1×1×1 Γ-centered k-point grid for binding energy calculations. Atomic partial charges on the perovskite surfaces were calculated using the Repeating Electrostatic Potential Extracted Atomic Charges (REPEAT) Method^[7]^ This technique fits atomic charges to reproduce the electrostatic potential obtained from periodic DFT calculations, yielding electrostatic potential (ESP)-derived atomic charges.

**
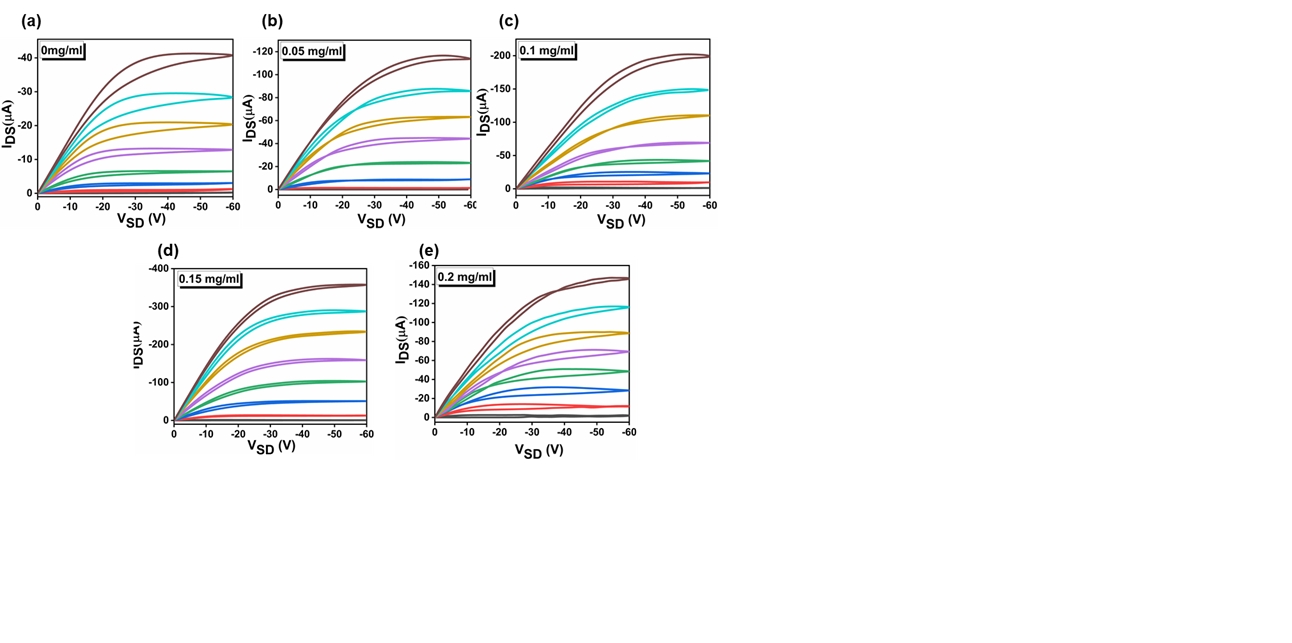
**

**Figure S2. (a-e)** Output characteristics for various concentration of BDTD in Sn-Pb based perovskite TFTs.

**

**

**Figure S3.** Transfer characteristics for various concentration of BDTD in Sn-Pb based perovskite TFTs.

**Table S1.** *V_th_*, *μ_h_* (maximum and average values), and *I_on_/I_off_* ratio for various concentration of BDTD in Sn-Pb based perovskite TFTs.

| **BDTD Amount (mg)** | ***V_th_***  **(V)** | ***µ_h_***  **(cm^2^ v^-1^s^-1^)** | ***V_DS_***  **(V)** | **on/off Ratio (*I_on_/I_off_*)** |
| --- | --- | --- | --- | --- |
| **0** | 10.79 | 0.37  (0.35) | -40 | 3.1 × 10^4^ |
| **0.05** | 10.19 | 0.81  (0.76) | -40 | 4.1 × 10^4^ |
| **0.10** | 10.10 | 1.7  (1.65) | -40 | 8.1 × 10^4^ |
| **0.15** | 9.90 | 4.1  (3.95) | -40 | 1.8 × 10^5^ |
| **0.20** | 11.1 | 2.95  (1.85) | -40 | 5.2 × 10^4^ |

**Supplementary Note 1: Comparison of FET performance with reported literature.**

The maximum field-effect mobility of 4.1 cm²/Vs achieved in this study for the MA₀.₄FA₀.₆(PbSn)₀.₅I₃ perovskite composition ranks among the highest reported for perovskite FETs, including both 2D and 3D perovskite-based devices, as summarized in **Table S2.**

**Table S2.** Critical performance metrics of 3D perovskite FETs documented in the literature.

| **Perovskite composition** | **Polarity** | **Mobility**  **(cm^2^/Vs)** | ***I_on_/I_off_***  **ratio** | **Ref.** |
| --- | --- | --- | --- | --- |
| **CsSnI_3_** | p | 50 | 5.0 x 10^7^ | ^[8]^ |
| **MaSnI_3_** | p | 19.6 | 2.2 x 10^7^ | ^[9]^ |
| **MAPbI_3_** | p | 23.2 | 5 x 10^3^ | ^[10]^ |
| **PEA_2_SnI_4_** | p | 15 | 4.0 x 10^5^ | ^[11]^ |
| **Cs_0.15_FA_0.85_Pb_0.5_Sn_0.5_I_3_** | p | 5.4 | 8.8 x 10^5^ | ^[12]^ |
| **Ma_0.4_Fa_0.6_(PbSn)_0.5_I_3_** | p | 0.7 | >10^3^ | ^[12]^ |
| **Ma_0.4_Fa_0.6_(PbSn)_0.5_I_3_**  **With additive** | p | 4.1 | 1.8 x 10^5^ | This work |
| **MAPbI_3_** | p | 4.7 | 6.7 x 10^3^ | ^[13]^ |
| **MAPbBr_3_** | p | 3.6 | 2.5 x 10^3^ | ^[13]^ |
| **MAPbCl_3_** | p | 3.8 | 3.8 x 10^4^ | ^[13]^ |
| **PEA_2_SnI_4_** | p | 2.6 | 6 x 10^5^ | ^[14]^ |
| **PEA_2_SnI_4_** | p | 0.62 | 1.3 x 10^4^ | ^[15]^ |
| **FAPb_0.5_Sn_0.5_I_3_** | p | 0.007 | 4.5 x 10^2^ | ^[16]^ |
| **(PEA, FA)SnI_3_** | p | 0.21 | 2 x 10^3^ | ^[17]^ |

**
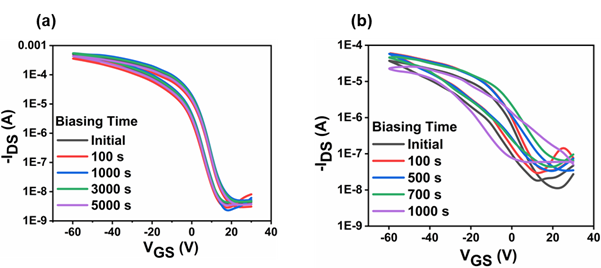
** **Figure S4.** **(a)** Bias-stress stability measurements were performed on MA₀.₄FA₀.₆(PbSn)₀.₅I₃ under two conditions: (a) with the optimized additive and **(b)** without the additive, under negative bias stress for varying durations. The measurements were conducted using a gate-to-source voltage (V_GS_) of -60 V and a drain-to-source voltage (V_DS_) of -40 V.

**

**

**Figure S5.** XRD pattern of perovskite composition MA_0.4_FA_0.6_(SnPb)_0.5_I_3_ precursor with different amount of additive.





**Figure S6.** FWHM of the (100) peak for perovskite films.

**
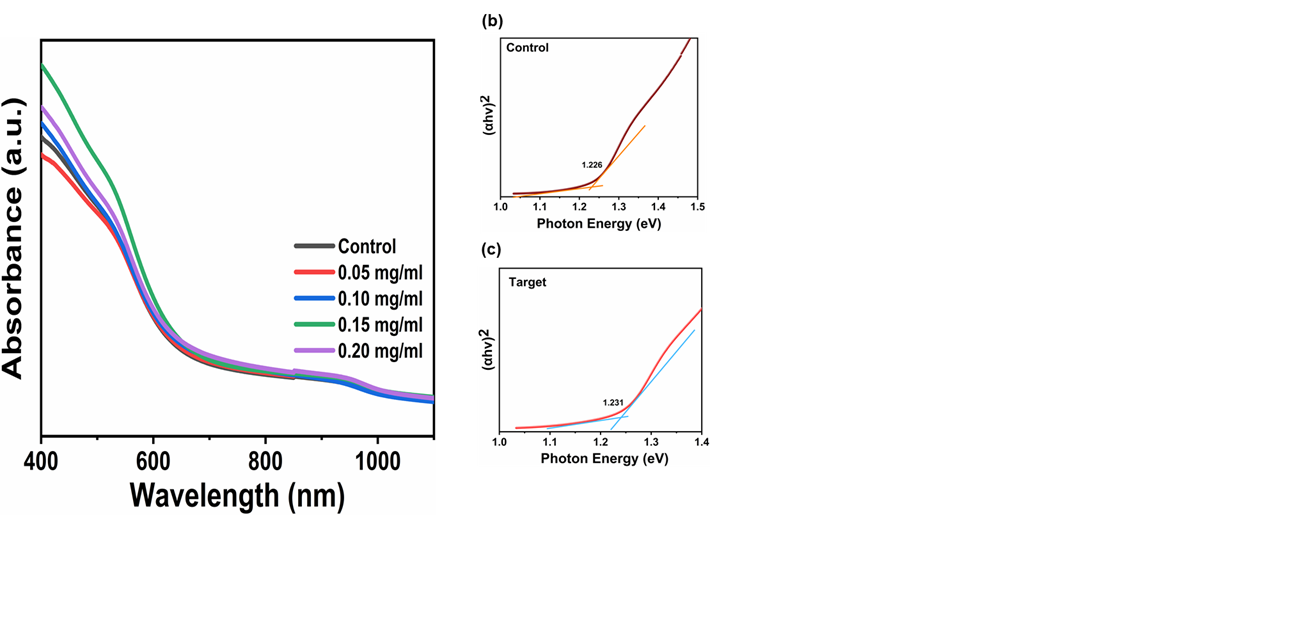
**

**Figure S7. (a)** UV–vis absorption spectra with different amount of additive. Tauc plots of **(b)** control and **(c)** target perovskite films.

**
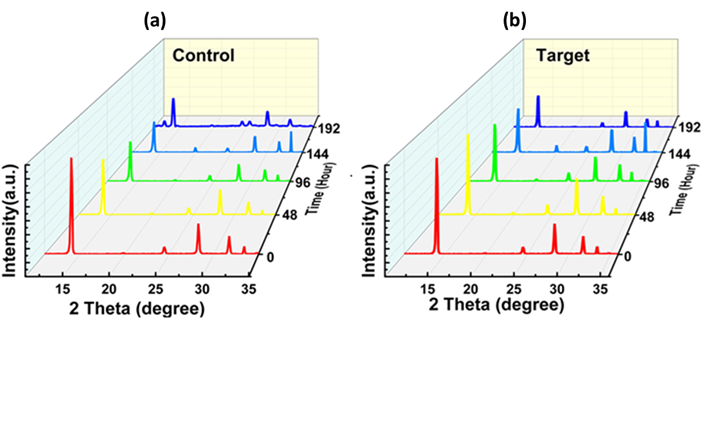
**

**Figure S8.** XRD patterns of **(a)** control and **(b)** target perovskite films.

**
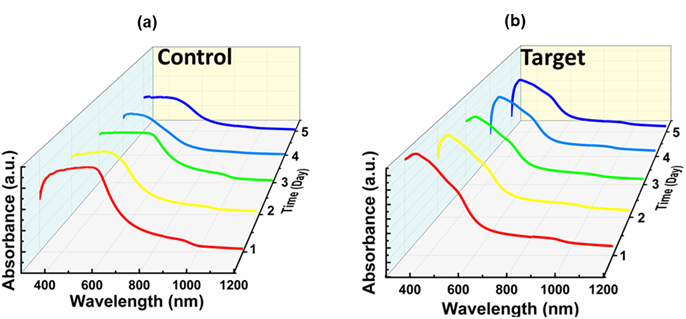
**

**Figure S9.** UV-vis absorption spectra of **(a)** control and **(b)** target perovskite films.

**
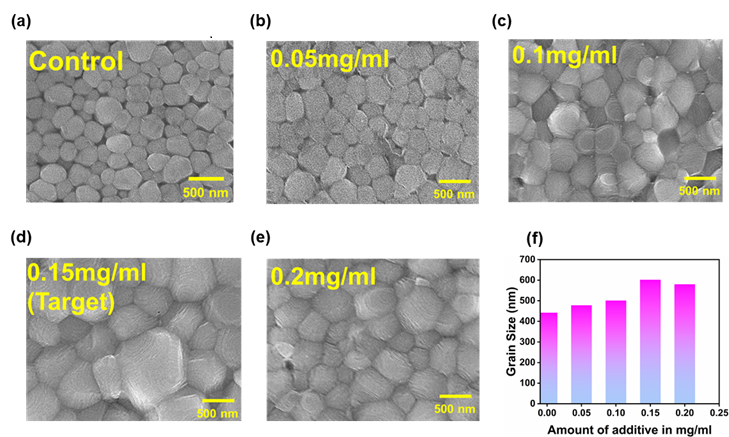
**

**Figure S10. (a-e)** Scanning electron microscopy (SEM) images of Sn-Pb mixed perovskite films with varying BDTD additive concentrations. **(f)** Grain size distribution at different additive concentrations.

**Table S3:** Scanning electron microscopy (SEM) images of Sn-Pb mixed perovskite films with varying BDTD additive concentrations .

| **Perovskite Composition**  **MA_0.4_FA_0.6_(SnPb)_0.5_I_3_** | **Mean Grain Size (nm)** | **Median Grain Size (nm)** |
| --- | --- | --- |
| Control | 455 ± 10 | 442 |
| With 0.05mg/ml additive | 467 ± 10 | 478 |
| With 0.1mg/ml additive | 510 ± 6 | 501 |
| With 0.15mg/ml additive | 610 ± 7 | 602 |
| With 0.2mg/ml additive | 580 ± 10 | 572 |

**
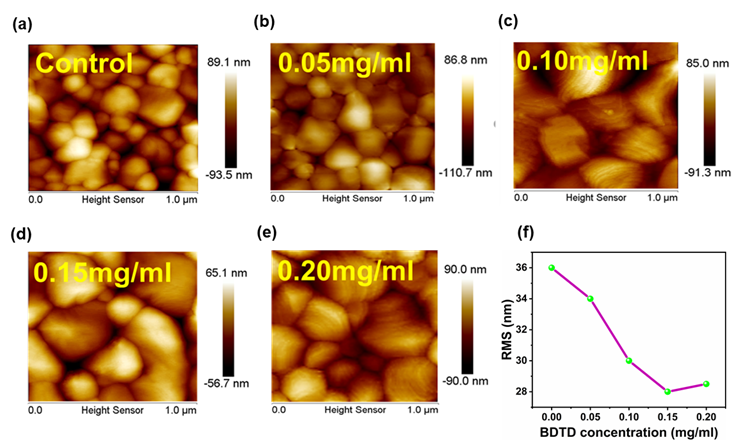
**

**Figure S11. (a-e)** Atomic force microscopy (AFM) images of Sn-Pb mixed perovskite films with different BDTD additive concentrations, and **(f)** R.M.S. values for films with varying BDTD additive concentrations.


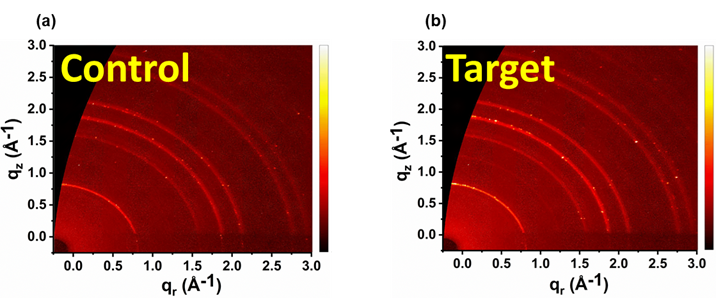


**Figure S12.** GIWAX patterns of **(a)** control and **(b)** target perovskite films.

**

**

**Figure S13.** Out-of-plane linecut profiles of the GIWAXS patterns.

**

**

**Figure S14.** FTIR spectra of BDTD, perovskite and BDTD-perovskite adduct.

**
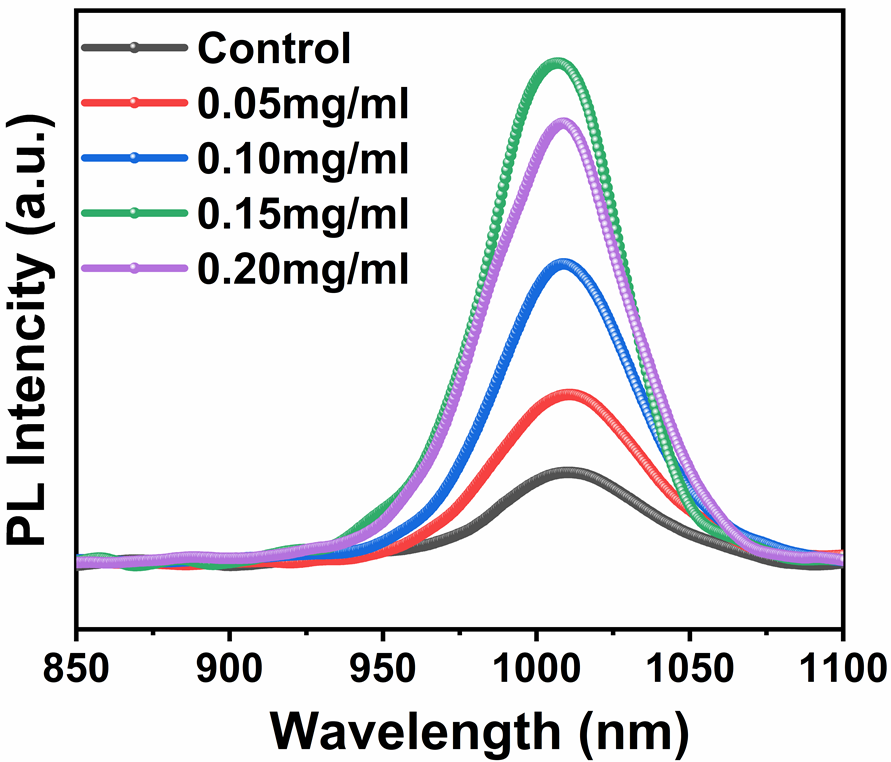
**

**Figure S15.** Photoluminescence (PL) spectra of Sn-Pb mixed perovskite films with varying BDTD additive concentrations.

**Table S4.** The deconvoluted XPS peaks of the newly synthesized control and target Sn-Pb mixed perovskite films.

|  | **3d_3/2_ Sn^2+^** | **3d_3/2_ Sn^4+^** | **3d_5/2_ Sn^2+^** | **3d_5/2_ Sn^4+^** |
| --- | --- | --- | --- | --- |
| **Control** | 486.60 eV | 487.50 eV | 495.0 eV | 495.80 eV |
| **Target** | 486.60 eV | 487.40 eV | 495.10 eV | 495.80 eV |

**
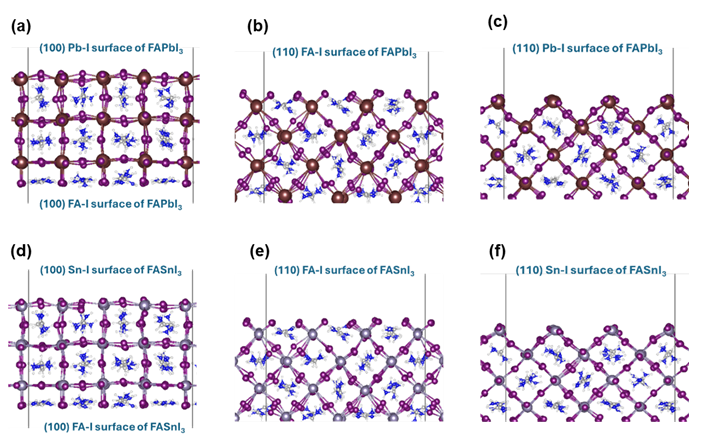
**

**Figure S16.** DFT-calculated geometrically optimized structures of the M-I and FA-I terminated surfaces of FAMI₃ on the (100) and (110) crystallographic facets.

**
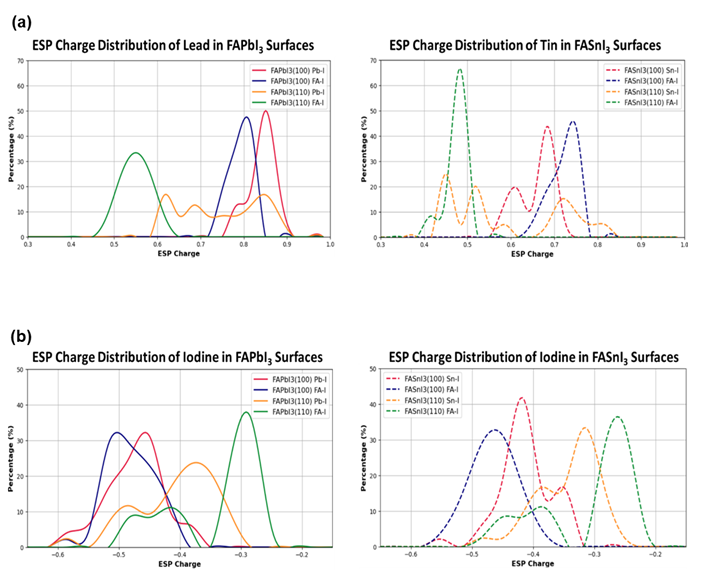
**

**Figure S17.** Distribution of ESP-derived atomic charges for **(a)** lead, tin and **(b)** iodine atoms in FAMI₃ across different surface terminations.

**Supplementary Note 2: Surface treatment of Perovskite film.**

A recent study has indicated that interface defects limit the further enhancement of power conversion efficiency (PCE) and the stability of perovskite solar cells (PSCs). In this work, we propose a strategy using tetrabutylammonium hexafluorophosphate (TBAPF₆) to passivate surface defects in perovskite films. Experimental results confirm that TBAPF₆ is uniformly distributed across the perovskite film and effectively passivates Pb²⁺ defects on the top surface. Additionally, the long alkyl chains in TBAH provide a protective barrier, reducing moisture-induced degradation of the perovskite layer.^[18]^ As a result, both the mobility and ON/OFF ratio of the perovskite thin-film transistor are significantly improved.

**
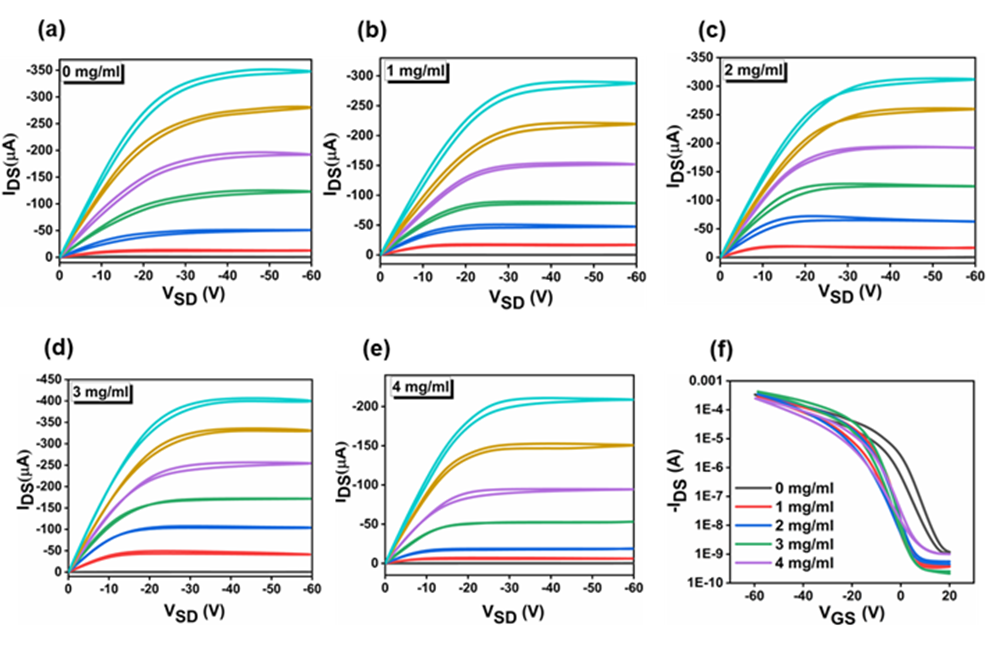
**

**Figure S18.** Output characteristics for different concentration of passivation material (tetrabutylammonium hexafluorophosphate ((TBAPF₆)) in Sn-Pb based perovskite.

**
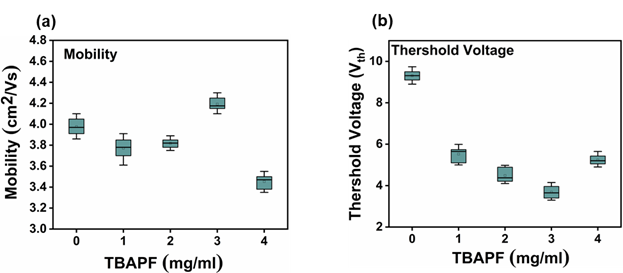
**

**Figure S19.** Mobility and threshold voltage with various amount of passivation material.

**Table S5.** *V_th_*, *µ_h_*, (maximum and average values), and *I_on_/I_off_* ratio for Sn-Pb perovskite with surface passivation using various concentrations of TBAPF₆.

| **Amount of passivation material TBAPF_6_** | **V_th_**  **(V)** | **Hole Mobility** **(µ_h_)**  **(cm^2^ v^-1^s^-1^)** | **V_DS_**  **(V)** | **on/off Ratio**  **(*I_on_/I_off_*)** |
| --- | --- | --- | --- | --- |
| **0** | 9.89  (10.1) | 4.1  (3.85) | -40 | 3.1 × 10^5^ |
| **1 mg/ml** | 4.7  (4.95) | 3.71  (3.66) | -40 | 2.1 × 10^6^ |
| **2 mg/ml** | 4.6  (4.80) | 3.8  (3.75) | -40 | 4.3 × 10^6^ |
| **3 mg/ml** | 3.6  (3.75) | 4.2  (4.15) | -40 | 5.8 × 10^6^ |
| **4 mg/ml** | 5.1  (5.30) | 3.4  (3.36) | -40 | 1.1 × 10^6^ |

**Reference**

1. Kresse, G. and J. Furthmüller, *Efficiency of ab-initio total energy calculations for metals and semiconductors using a plane-wave basis set.* Computational materials science, 1996. **6**(1): p. 15–50.

2. Kresse, G. and J. Furthmüller, *Efficient iterative schemes for ab initio total-energy calculations using a plane-wave basis set.* Physical review B, 1996. **54**(16): p. 11169.

3. Kresse, G. and D. Joubert, *From ultrasoft pseudopotentials to the projector augmented-wave method.* Physical review b, 1999. **59**(3): p. 1758.

4. Perdew, J.P., K. Burke, and M. Ernzerhof, *Generalized gradient approximation made simple.* Physical review letters, 1996. **77**(18): p. 3865.

5. Grimme, S., et al., *A consistent and accurate ab initio parametrization of density functional dispersion correction (DFT-D) for the 94 elements H-Pu.* The Journal of chemical physics, 2010. **132**(15).

6. Monkhorst, H.J. and J.D. Pack, *Special points for Brillouin-zone integrations.* Physical review B, 1976. **13**(12): p. 5188.

7. Campañá, C., B. Mussard, and T.K. Woo, *Electrostatic potential derived atomic charges for periodic systems using a modified error functional.* Journal of Chemical Theory and Computation, 2009. **5**(10): p. 2866–2878.

8. Liu, A., et al., *High-performance inorganic metal halide perovskite transistors.* Nature Electronics, 2022. **5**(2): p. 78–83.

9. Zhu, H., et al., *High-performance hysteresis-free perovskite transistors through anion engineering.* Nature communications, 2022. **13**(1): p. 1741.

10. Jana, S., et al., *Toward stable solution-processed high-mobility p-type thin film transistors based on halide perovskites.* ACS nano, 2020. **14**(11): p. 14790–14797.

11. Matsushima, T., et al., *Solution-Processed Organic-Inorganic Perovskite Field-Effect Transistors with High Hole Mobilities.* Advanced Materials (Deerfield Beach, Fla.), 2016. **28**(46): p. 10275–10281.

12. Senanayak, S.P., et al., *Charge transport in mixed metal halide perovskite semiconductors.* Nature Materials, 2023. **22**(2): p. 216–224.

13. Yu, W., et al., *Single crystal hybrid perovskite field-effect transistors.* Nature communications, 2018. **9**(1): p. 5354.

14. Mitzi, D.B., et al., *Hybrid field‐effect transistor based on a low‐temperature melt‐processed channel layer.* Advanced Materials, 2002. **14**(23): p. 1772–1776.

15. Kagan, C.R., D.B. Mitzi, and C.D. Dimitrakopoulos, *Organic-inorganic hybrid materials as semiconducting channels in thin-film field-effect transistors.* Science, 1999. **286**(5441): p. 945–947.

16. Bowman, A.R., et al., *Microsecond carrier lifetimes, controlled p-doping, and enhanced air stability in low-bandgap metal halide perovskites.* ACS energy letters, 2019. **4**(9): p. 2301–2307.

17. Shao, S., et al., *Field‐effect transistors based on formamidinium tin triiodide perovskite.* Advanced Functional Materials, 2021. **31**(11): p. 2008478.

18. Xia, Z., et al., *Improving the efficiency and stability of perovskite solar cell through tetrabutylammonium hexafluorophosphate post-treatment assisted top surface defect passivation.* Solar Energy Materials and Solar Cells, 2022. **248**: p. 112011.
